# Impact of children born with low birth weight on stunting and wasting in Sindh province of Pakistan: a propensity score matching approach

**DOI:** 10.1038/s41598-021-98924-7

**Published:** 2021-10-07

**Authors:** Faisal Abbas, Ramesh Kumar, Tahir Mahmood, Ratana Somrongthong

**Affiliations:** 1 Department of Economics, School of Social Sciences and Humanities (S3H), National University of Sciences and Technology (NUST), Sector H-12, Islamabad, 44000 Pakistan; 2 Department of Public Health, Health Services Academy, Islamabad, Pakistan; 3 Department of Economics, University of Chitral, Chitral, Pakistan; 4 College of Public Health Sciences, Chulalongkorn University, Bangkok, Thailand

**Keywords:** Health care, Medical research

## Abstract

Low Birth Weight (LBW) is considered as a major public health issue and leading cause of neonatal death. Almost one in four newborns are reported as underweight in Pakistan. Children born with low birth weight are highly vulnerable to develop diseases and death and/or remain undernourished (i.e., stunted and wasted). This study determines the LBW newborns are more prone to develop stunting and wasting in province of Sindh, Pakistan. Moreover, regression-based estimation of the impact of LBW on the child health outcomes of under five years of age, may be prone to selection bias because of the nature of non-experimental data set, thus, propensity score matching methods are used in this study. Data for this study was used from Multiple Indicators Cluster Survey (MICS-2014). MICS is a two-stage, stratified cluster sampling household level data covering urban and rural areas and consists of 19,500 households from five administrative divisions and 28 districts of Sindh province of Pakistan. The total sample size of children less than five years of age after cleaning the data are 7781, of which 2095 are LBW having birth weight categorized as “smaller than average and very small” and 5686 are normal birth weight (NBW) having birth weight very large, larger than average, and average. This study employed propensity score matching (PSM) regression methods to understand whether the children born as low birth weight are more prone to stunting and wasting and/or both. In province of Sindh, moderate wasting children under five years were 21%, severe wasting 6% and both wasting and stunting 10%. The propensity score results are shown significant in all groups. Specifically, all four types of PSM methods confirm a significant difference in the potential outcome variables—meaning that a child born with LBW has a significant adverse effect on the potential child health outcome variables (stunting, wasting and both). Thus, the propensity score matching findings confirm a significant and adverse effect of LBW on potential health outcomes of under five children. Similarly, low birth weight children are significantly more likely to be moderately wasted (OR = 1.5, CI = 1.3–1.6) and severely wasted (OR = 1.6, CI = 1.3–2.0) and both (stunted and wasted, OR = 2.0, CI = 1.7–2.3) as compared to children with normal birth weight. Male children, if born with low birth weight, are significantly more likely to be moderately wasted (OR = 1.3, CI = 1.1–1.5) and both (wasted and stunted, OR = 1.3, CI = 1.1–1.5) than girls. This large data analysis finding proved that the LBW newborns are on higher risk to develop wasting and stunting in Pakistan.

## Introduction

About 20 million of babies are born with low birth weight (LBW) worldwide^1^. Above 80% neonatal deaths were reported in LBW newborns including preterm and term small for gestational age^2^. About half of childhood deaths occur due to malnutrition and around 144 million children below five years of age were reported stunted and 47 million are reported as wasting globally^3^. Furthermore, it has been reported about 6 million children were stunted and wasted^4^. LBW is highly prevalent in particularly south Asian countries like, India, Pakistan and Bangladesh^2,5,6^.

LBW and preterm births are main causes of infant mortality that accounts for 30% child deaths during their first 28 days of life^7^. Hence, LBW is considered as a major public health issue and leading cause of neonatal death with multiple health related adverse effects. LBW is an important health indicator for survival of babies born with weight less than 2500 g^7^. The children are on higher risk of developing diseases and deaths due to their low birth weight during childhood period^8^.

Past research has revealed that the factors like length of gestation, parity, perinatal care, parent’s education level, low socio-economic status, maternal anemia, short stature of the mother, birth spacing and infections are strongly associated with LBW^9–13^. Other environmental factors like good personal hygiene and improved sanitation could reduce LBW incidence and decrease child mortality by 14–31%^14^. LBW has long term consequences on postnatal development, increased risk of respiratory distress, hypoglycemia, polycythemia, mental disabilities, cerebral palsy, food intake issues, sensory and vision dysfunction and later in life this can develop cardiovascular diseases and diabetes^15–18^.

According to Pakistan demographic health survey, (23%) of children under five year of age were reported as underweight^19^. The prevalence of stunting has dropped by 11% and wasting has declined, from 45 to 38%, during last 5 years^19,20^. Sindh province has reported highest prevalence of stunting and wasting among all the province of Pakistan^19^. Data shows four out of ten children below five-year age are underweight (42%) and almost half (48%) are stunted and (15%) children reported wasted in Sindh province^21^.

LBW and its immediate consequences on child health outcomes particularly stunting and wasting are not well documented empirically in Pakistan. Hence, the current study tries to fill this gap. Moreover, the study will come up with the empirical results, providing guidelines to the policy makers. The impact evaluation of LBW forms the foundation of public policy analysis. During the last two decades, impact evaluation through PSM has gained tremendous attention in applied econometrics. The current study utilizes the propensity score matching method to estimate the impact of LBW on the malnourished under five years of children for the case of Sindh, Pakistan. The data comes from the observational studies, which are none, randomize. The presence of such none randomize studies confronts with the problem of selection bias. To ward off or reduces the chances of such bias, a proposed PSM method^22^, is extensively used in economic policy intervention and in medical trial^23^.

## Methods

### Participants and study settings

The Multiple Indicator Cluster Survey (MICS) is an international household survey programme developed by the United Nations Children’s Fund (UNICEF). In Sindh, between January and August 2014, MICS collected household-level data to analyse the health of women and children, while also employing anthropometric (nutrition) measures and education measures among other key indicators. The two-stage stratified cluster sampling MICS data covered rural and urban areas of all 5 divisions and 28 districts of the province. Enumeration Blocks (EB) in urban areas and villages in rural areas were the primary sampling units (PSU). Households were considered as secondary sampling units, and from each PSU, 20 households were selected with a random start. The total sample consisted of 19,500 households with a response rate of 94%^21^.

### Variables

The analysis incorporates moderate wasting, severe wasting and combination of both wasting and stunting as outcome variables; whereas, the main explanatory variable is the weight of the child at birth. The birth indicator is generated as a binary variable by categorizing the original question (size of child at birth) such as very large, larger than average, average as “*normal birth weight (NBW)”* and smaller than average and very small as “*low birth weight (LBW)”*. The total sample size after cleaning the data are 7781, out of which 2095 under five years of age are LBW *(treated/LBW group)* and 5686 *(untreated/NBW group)* are normal birth weight children. Many important covariates: that’s child specific characteristic-age, birth order, sex and health condition related variables (infection, pneumonia and diarrhea); mother specific characteristics-age, education, health condition and household characteristics-sanitation, wealth are also included in the analysis.

This study employs propensity score matching (PSM) regression methods. Because, the multivariate regression analysis relies on the modelling assumptions of linearity between the confounders and the odds of the outcome variables. However, the PSM eliminates the linearity assumptions and exhibits more empirical power than logistic regression^24^. Matching of children is done based on the covariates of both treated (LBW) and controlled (NBW) groups. These matching estimates indicate that conditional on a set of individual characteristics of the treatment or assignment is independent of potential outcomes. The PSM usually involves three steps. First, estimating the propensity score (here we looked at checking the balancing property based on covariate and the distribution of propensity score for each groups graphically). Second, matching the propensity score through four widely used methods used in the current study and third, making the impact analysis through match samples. A plethora of literature exists to matching the propensity score. However, widely used methods are, Nearest Neighbor Matching (NNM), Radius Matching (RM), Kernel Matching (KM), and Stratification Matching (SM)^25^.

In the NNM each treated unit is matched with controlled unit with the nearest propensity score. With replacement each treated unit is compared with the nearest controlled group (NBW), hence minimizing the propensity score distance, leading to reduce the bias between the two groups. However, if the comparison group is small, the nearest neighbor matching suffers from the risk of bad matches. In this case, the RM comes to the rescue^26^. All cases in the comparison group with estimated propensity scores falling within radius are matched to the treated case. Another matching method is the KM that uses the weighted averages of all individuals in the control group (NBW) to construct the counterfactual outcome^27,28^. The weights are inversely proportional to the distance between the propensity scores of the treated and comparison group. All treated units are matched with a weighted average of all comparison units. Finally, the SM divides the observations into five equal intervals based on the propensity scores, and then the difference between the average outcomes of the treated (LBW) and controlled group (NBW) is obtained. Weights are applied across intervals to calculate the average treatment effect. It excludes observations in blocks where either treated or controlled units are absent.

The primary objective of the propensity score is not to analyze only the statistical properties of the parameters estimated, but also concern with the balancing properties of the covariates^26,29,30^. Once the propensity score matching is performed, the balancing test is used for the purpose of balancing property. Balancing property is commonly tested by three methods. (1) checking standard bias estimation, (2) before and after treatment, (3) testing the significance difference between the treated (LBW) and controlled group (NBW) by t test. In this study, we used two sample t test. The estimated model having low t values satisfies the balancing property^29^. Second, re-calculation for the matched sample of propensity score calculation for the treated (LBW) and controlled group (NBW), and comparing pseudo R^2^ before and after matching. Low value indicates fulfillment of balancing property. Finally, through stratification method, in which sample are divided into blocks. The balancing property is satisfied if the difference between the two groups is insignificant using t test^26^. The region of common support is to be defined after the balancing property fulfillment. In this common support region, the propensity score for both groups should be overlapped. Minima and maxima standard approach are utilized for the common support region. Higher than the maxima and lower than the minima value, the observations are dropped from the sample^31^. The greater overlap between the groups, the lesser the chance of the bias^28^. After the satisfaction of the above mentioned properties, next task is to perform matching through estimated propensity score and for matching this study employed PSM methodology.

Before, we delve into the PSM estimation, we will first check if there is a significant difference between the means of normal birth weight child and low birth weight child to set the stage for PSM between the treated (LBW) and comparison groups (NBW). To this end, we will compare the two groups via t-test. Mathematically, the t-test takes a sample from each of the two sets and establishes the problem statement by assuming a null hypothesis that the two means are equal.

### Ethics approval and consent to participate

This study is based on an analysis of cross sectional data available freely and publicly with all identifier information removed, no ethics approvals were required.

## Results

### Descriptive statistics

The total sample size were 7,781, out of which 2,095 under five years of age are low birth weight (LBW) *(treated/LBW group)* and normal birth weight (NBW) 5,686 *(untreated/NBW group)* were included in this study. Moderate wasting children were (21%), severe wasting (6%) and both wasting and stunting (10%) found under five years of age. More than half (55%) children were male and (19%) had their birth order more than one child. One-third (34%) of children were suffering from Diarrhoea and nearly half (46%) were reported ill with fever. About (77%) families were visited by lady health workers at their homes and received antenatal care during pregnancy. Less than half (43%) were delivered at hospital and (54%) mothers get tetanus toxoid injection. More than half (57%) children were living in the houses with good sanitation. The descriptive statistics of the variables are given in (Table 1).

**Table 1:** Mother’s socio-demographic and economic characteristics (n = 7781)-Full sample.

Variables	Mean	Standard deviation	Minimum	Maximum
Age	2.754	1.401	1	6
Gender	0.509	0.500	0 = female	1 = male
Birth order	0.192	0.394	0 = 1 child	1 = > 1 child
Child with Diarrhea	0.343	0.475	0 = No	1 = Yes
Child Ill with fever	0.465	0.499	0 = No	1 = Yes
Lady Health worker Visit	0.774	0.419	0 = No	1 = Yes
ANC	0.772	0.420	0 = No	1 = Yes
Hospital Delivery	0.430	0.495	0 = No	1 = Yes
Tetanus injection	0.542	0.498	0 = No	1 = Yes
Mother age	2.810	0.609	1	4
Mother education	1.716	1.224	1	5
House hold size	2.924	1.057	1	4
Sanitation Facility	0.577	0.494	0 = No	1 = Yes
Ethnicity	2.641	0.984	1	4
Wealth index	1.545	0.762	1	3
Region	0.371	0.483	0 = Rural	1 = Urban
Division	2.712	1.293	1	5

### Empirical results

The two-sample test of proportions results confirm a significant mean difference between the treated group (LBW) and control group ((NBW)). The mean difference findings are shown in (Table 2). This difference in mean manifests the use of propensity score. Moderate wasting among children less than five years of age was reported in treated (26%) and untreated (19%) with mean significant difference of (7%). Severe wasting was reported in treated (9%) and untreated (5%) with mean significant difference of (4%). However, both wasting and stunting were reported in treated (16%) and untreated (8%) with mean significant difference of (8%). Female children under age of 5 had observed (8%) significant difference as compared to male (7%) in this study. This shows that female children are more affected with LBW as compared to males. This significant gender difference might be due the different nutritional requirements and girls are less likely than boys to access basic services and have, on average, consistently lower development outcomes. Hence, we need to explore this difference by using this MICS data through gender sub-analysis approach.

**Table 2:** Two-sample test of proportions between LBW and NBW groups.

*Complete Sample*						
	Moderate Wasting (MW)		Severe wasting (SW)		Both (wasting, stunting)	
	Untreated	Treated	Untreated	Treated	Untreated	Treated
Mean	0.192	0.265	0.054	0.093	0.088	0.165
Difference	− 0.073***		− 0.038***		− 0.076***	
SE	0.010		0.006		0.007	
t-value	− 7.019		− 6.169		− 9.707	
N	5686	2095	5686	2095	5686	2095
**Female sample**						
Mean	0.217	0.282	0.050	0.097	0.1	0.182
Difference	− 0.065***		− 0.037***		− 0.082***	
SE	0.015		0.009		0.012	
t-value	− 4.231		− 4.000		− 6.937	
N	2940	1024	2940	1024	2940	1024
**Male sample**						
Mean	0.165	0.248	0.048	0.089	0.075	0.148
Difference	− 0.084		− 0.041		− 0.073	
SE	0.014		0.008		0.011	
t-value	− 5.976		− 4.835		− 6.974	
N	2746	1071	2746	1071	2746	1071

### Propensity score: checking balance

Before we start analyzing the data, we compared all of the confounders between the treated (LBW) and untreated (NBW). Listing shows how we can get the mean and standard deviation for each variable in the treated (LBW) and untreated (NBW). The output shows us that the LBW and NBW groups differ by about less than 1 standard deviation (SD). So, the LBW and NBW groups are more similar. The balancing property is satisfied for both groups male and female. The low level of t test indicates that the distribution of conditioning covariates does not differ between the treated (LBW) and controlled groups (NBW) (Appendix A).

**Figure 1:**
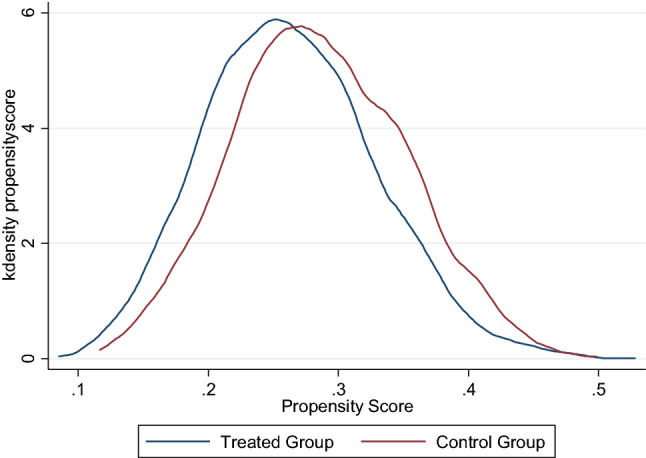
Distribution of propensity scores between treated (LBW) and untreated groups (NBW).

We can now look at the distributions of the propensity score in the treated (LBW) and the untreated (NBW) groups. The result is shown in Fig. 1, a much more normal distribution in both subgroups. Justify the assumptions of propensity score.

### Propensity score findings

The result of average treatment effect (ATE) using PSM methods is given in Table 3. The ATE measures the difference in mean of the outcome variables between units assigned to the LBW and NBW. Importantly, propensity scores solve the fundamental problem of causal inference. Specifically, it modifies the analysis by balancing the covariates (say age, sex, gender, health status etc.) between the LBW and NBW groups, then we can infer strong evidence that the difference in outcomes (stunting, wasting, both) is due to the LBW rather than these covariates. Its magnitude ranges between zero and one. The larger the propensity score, more likely individual of being treated. All the results are significant. For instance, if we look at the complete model findings, there is a significant difference depicted by all four different types of PSM methods, meaning that a child born with LBW has a significant adverse effect on the potential child health outcome variables (stunting, wasting and both). Similarly, for sub samples, the ATE values show a significant difference regarding the potential outcomes between the LBW and NBW groups for under five years of children.

**Table 3:** Average treatment effect of LBW under various methods of PSM on malnutrition.

Method	Complete Sample			Sub sample (Female)			Sub sample (Male)		
	MW	SW	Both	MW	SW	Both	MW	SW	Both
**Nearest neighbor matching method**									
ATE	0.079	0.046	0.075	0.078	0.051	0.081	0.110	0.039	0.078
N. Treated	2095	2095	2095	1024	1024	1024	1071	1071	1071
N. Control	1636	1636	1636	839	839	839	804	804	804
SE	0.016	0.006	0.012	0.022	0.009	0.019	0.019	0.007	0.014
t-stat	5.081	7.412	6.265	3.467	5.881	4.201	5.719	5.907	5.745
**Radius matching method**									
ATE	0.069	0.036	0.075	0.066	0.035	0.084	0.077	0.038	0.069
N. Treated	2095	2095	2095	1024	1024	1024	1071	1071	1071
N. Control	5666	5666	5666	2919	2919	2919	2724	2724	2724
SE	0.011	0.007	0.009	0.016	0.010	0.013	0.015	0.010	0.012
t-stat	6.277	5.156	8.350	4.067	3.399	6.276	5.078	3.934	5.676
**Kernel matching method**									
ATE	0.069	0.036	0.075	0.065	0.035	0.083	0.075	0.038	0.067
N. Treated	2095	2095	2095	1024	1024	1024	1071	1071	1071
N. Control	5666	5666	5666	2919	2919	2919	2724	2724	2724
SE	0.009	0.010	0.005	0.005	0.009	0.009	0.010	0.011	0.007
t-stat	7.654	3.618	16.131	14.103	3.841	8.897	7.659	3.393	10.069
**Stratification method**									
ATE	0.067	0.036	0.073	0.059	0.033	0.081	0.075	0.040	0.065
N. Treated	2095	2095	2095	1024	1024	1024	1071	1071	1071
N. Control	5666	5666	5666	2913	2913	2913	2724	2724	2724
SE	0.012	0.010	0.009	0.017	0.005	0.013	0.019	0.010	0.009
t-stat	5.811	3.470	8.517	3.389	6.382	6.047	3.846	4.022	7.165

MW (Moderate wasting); SW (Severe wasting); both (moderate wasting and moderate stunting).

Table 4 presents the results of the multivariate logistic regression analyses. Low birth weight children are significantly more likely to be moderately wasted (OR = 1.5, 1.3–1.6) and severely wasted (OR = 1.6, 1.3–2.0) and both (stunted and wasted, OR = 2.0, 1.7–2.3) as compared to children with normal birth weight. In the first two years, the child weight for height (moderate and severe stunting) progressively improves after two years of age. This weight gain could be due to the nutritional fulfillment as the children starts his/her own feeding practices. This also confirms that the low weight of children is due to the nutritional deficiencies and poor health condition of their mother. Height for age (stunting) of a child is significantly lagged behind relatively in the first two years of the child. The odds for 24–35 months (3rd year) are 1.65, that is lower than the odds for 12–23 months (2nd year) are 2.68, compared the reference category of child 0–5 months. Male children are significantly more likely to be moderately wasted (OR = 1.3, 1.1–1.5) and both (wasted and stunted, OR = 1.3, 1.1–1.5) than girls. Children whose birth order is second is significantly likely to be moderate wasted (OR = 1.33, 1.1–1.4) and both (stunted and wasted) (OR = 1.3, 1.1–1.5). Children with no diarrhea are significantly less likely to be stunted and wasted both as compared to those having an episode of diarrhea (OR = 1.3, 1.0–1.5). Children with fever are significantly more likely to be moderately (OR = 1.3, 1.0–1.5) and severely wasted (OR = 1.6, 1.3–2.0) and both (wasted and stunted, OR = 1.3, 1.3–2.0) as compared to those having no fever. Mothers of those children who got antenatal care are significantly less likely to be moderately wasted (OR = 0.7, 0.6–0.8) and both (wasted and stunted, OR = 0.7, 0.6–0.8) as compared to those who did not received ANC. Those children born in hospital are significantly less likely to be moderately wasted (OR = 0.8, 0.7–0.9) as compared to those who borne outside hospital. Mothers who got tetanus injection are significantly less likely to be moderately (OR = 0.8, 0.7–0.9) and severely wasted (OR = 0.8, 0.6–0.9) and both (wasted and stunted, OR = 0.7, 0.6–0.8) as compared to those who had not.

**Table 4:** Multivariable regression analysis of moderate, severe wasting and both with other variables.

Variables	Moderate wasting OR (95% CI)	Severe wasting OR (95% CI)	Wasting and Stunting OR (95% CI)
**Birth weight (Reference Category Normal)**			
Low birth weight	1.496*** (1.32,1.69)	1.661*** (1.36,2.01)	2.023*** (1.73,2.36)
**Child age group (Reference Category 0–5 months)**			
6–11	0.917 (0.77,1.07)	0.713** (0.55,0.92)	1.538*** (1.19,1.99)
12–23	0.959 (0.82,1.11)	0.680*** (0.17,0.48)	2.682*** (2.14,3.36)
24–35	0.449*** (0.34,0.59)	0.288*** (0.17,0.49)	1.655** (1.17,2.35)
36–47	0.439*** (0.33,0.58)	0.116*** (0.05,0.25)	1.378 (0.96,1.97)
48–59	0.373*** (0.27,0.51)	0.177*** (0.09,0.35)	1.071 (0.71,1.62)
**Gender (Reference Category female)**			
Male	1.335*** (1.19,1.50)	1.185 (0.98,1.43)	1.333*** (1.15,1.55)
**Birth order (Reference Category one child)**			
More than one child	1.291*** (1.13,1.48)	1.135 (0.91,1.41)	1.325** (1.11,1.58)
**Diarrhoea (Reference Category No diarrhea)**			
Diarrhoea	1.037 (0.92,1.17)	0.977 (0.80,1.20)	1.280** (1.09,1.50)
**Fever (Reference Category No fever)**			
Ill with fever	1.323*** (1.17,1.50)	1.664*** (1.36,2.03)	1.511*** (1.29,1.78)
**LHW visit (Reference Category No visit)**			
LHW	0.992 (0,86,1.15)	0.869 (0.69,1.09)	0.854 (0.71,1.03)
**ANC (Reference Category No ANC)**			
ANC	0.715*** (0.62,0.82)	0.865 (0.69,1.08)	0.743*** (0.62,0.89)
**Hospital Delivery (Reference Category No hospital delivery)**			
Hospital Delivery	0.854* (0.75,0.97)	0.936 (0.76,0.92)	0.921 (0.78,1.09)
**Tetanus injection (Reference Category No tetanus injection)**			
Tetanus injection	0.852** (0.76,0.96)	0.817* (0.67,0.99)	0.742*** (0.63,0.87)
**Mother age (Reference Category age < 18 years)**			
18–24	2.725** (1.29,5.75)	8.365* (1.15,60.90)	2.595 (0.93,7.26)
25–35	3.133** (1.49,6.58)	7.969* (1.10,57.89)	2.493 (0.90,6.94)
36 +	3.297** (1.54,7.05)	9.184* (1.25,67.77)	2.124 (0.75,6.06)
**Mother education (Reference Category no education)**			
Primary	0.958 (0.81,1.14)	0.692* (0.51,0.94)	0.744* (0.59,0.95)
Middle	0.584** (0.42,0.82)	0.461* (0.24,0.88)	0.856 (0.55,1.32)
Secondary	0.652** (0.49,0.88)	0.618 (0.37,1.03)	0.649 (0.42,1.00)
Higher	0.726* (0.54,0.98)	0.288*** (0.15,0.57)	0.340*** (0.19,0.62)
**Household size (Reference Category 3–4 members)**			
5–6 members	0.826 (0.68,1.01)	0.760 (0.55,1.05)	0.828 (0.64,1.07)
7–8 members	0.869 (0.71,1.07)	0.761 (0.55,1.06)	0.832 (0.64,1.08)
8 + members	0.847 (0.70,1.02)	0.846 (0.63,1.14)	0.769* (0.60,0.98)
**Sanitation (Reference Category unimproved)**			
Improved	0.818** (0.71,0.95)	0.839 (0.67,1.06)	0.967 (0.80,1.16)
**Language (Reference Category urdu)**			
Sindhi	0.640** (0.49,0.84)	0.929 (0.55,1.58)	0.742 (0.48,1.15)
Saraikai	0.733* (0.54,0.10)	1.214 (0.69,2.13)	0.877 (0.55,1.40)
Others	0.680** (0.52,0.10)	0.964 (0.57,1.63)	0.739 (0.48,1.14)
**Wealth index (Reference Category poor)**			
Middle	0.794 (0.66,0.96)	0.654 (0.47,0.91)	0.679** (0.53,0.87)
Rich	0.890 (0.69,1.15)	1.065 (0.69,1.64)	0.531** (0.36,0.79)
**Area (Reference Category Rural)**			
Urban	1.296*** (1.12,1.50)	1.297* (1.02,1.28)	1.232* (1.01,1.45)
**Division (Reference Category Larkana)**			
Sukkar	1.104 (0.92,1.33)	0.997 (0.74,1.34)	0.957 (0.75,1.22)
Hyderabad	1.682*** (1.42,1.99)	1.361* (1.03,1.79)	1.529*** (1.23,1.90)
Mirpur Khas	1.935*** (1.60,2.34)	1.645** (1.22,2.22)	1.905*** (1.50,2.42)
Karachi	1.065*** (0.81,1.41)	0.975 (0.60,1.59)	0.780 (0.51,1.19)
Constant	0.146*** (0.06,0.33)	0.0139*** (0.00,0.11)	0.0343*** (0.01,0.11)

Mothers’ education is a significant covariate of moderate wasting. Compared to no education reference category, children of highly educated mother are significantly less likely to be moderately wasted (OR = 0.7, 0.5–0.9) and severely wasted (OR = 0.2, 0.1–0.5) and both (wasted and stunted, OR = 0.3, 0.1–0.6) as compared to no education. Whereas children of Primary (OR = 0.6, 0.5–0.9) and Middle school completed mothers (OR = 0.4, 0.2–0.8) are significantly less likely than no educated mothers to be severely wasted. Those children living in houses with improved sanitation are significantly less likely to be moderately wasted (OR = 0.8, 0.7–0.9) as compared to those who don’t have improved sanitation at their houses. Parents of children with middle (OR = 0.6, 0.5–0.8) and rich (OR = 0.5, 0.3–0.7) wealth index are significantly less likely to be both stunted and wasted as compared to those who were poor. Families of children with speaking Sindhi (OR = 0.6, 0.4–0.8), Saraiki (OR = 0.7, 0.5–0.1) and others (OR = 0.6, 0.5–0.1) are significantly less likely to be moderately wasted as compared to those who speak Urdu language.

## Discussions

This study analysed the impact of LBW on the child health outcomes including stunting, wasting and combination of both using matched pairs from the MICS data among children less than five years of age while adjusting for other confounders. This data also provided an ideal opportunity to control a large set of confounding factors when using the PSM approach. Moreover, LBW newborns are on high risk to develop wasting and stunting in Pakistan. This research has highlighted that the socioeconomic, demographic and personal factors are accountable for LBW likewise, those who had birth order more than one, with diarrhoea, male child and delivered at home were on high risk to develop LBW. PSM analysis can only be as good as the quality and the completeness of potential confounding variables that are at the disposal of the researcher. Study supports our finding with strong association of LBW children with family size, income and number of children in their family^32^. National Nutrition Survey of Pakistan data shows that two fifth of children below five years are stunted and one fifth is wasted in Pakistan and government needs to prioritise this public health problem and special interventions are also required^5,33^. PSM approach could provide reliable and efficient methods to control for covariates or potential confounding variables. Data from demographic and health survey and Multi-indicator Cluster Surveys datasets from 84 countries findings are consistent with our analysis and shows the prevalence for being wasted, stunted, and concurrently wasted and stunted among children less than five years^4^. Similar survey findings revealed that child age, birth order, education of parents, poor sanitation facilities and poverty are associated significantly with the likelihood of moderate and severe stunting in one of the province of Pakistan^6,33^. Study supported our findings that the families with choice of male babies could results malnourished and underweight babies in their families^34^. Another research has also similar with our results that the multiple factors like; babies living with large family size with sharing common room and illiteracy are on higher risk to develop LBW^35^. Local research has proved that the boys are more stunted as compare to girls and diarrhoea was also associated with LBW^36^. Research also found that a birth spacing less than two years was a major cause of stunting in the similar population where this study analysis was done^37^. Furthermore, recent multicounty analysis suggested that a quality antenatal care services could prevent LBW among their babies significantly^38^. Evidence shows that exclusive breastfeeding up to six months, complementary feeding for two years of age and early initiation of breast milk within hour of birth results in adequate growth among low birth weight babies and lower the risk of newborn deaths^39,40^. Environmental factors as good personal hygiene and improved sanitation could reduce LBW incidence and decrease child mortality by 14–31%^14^. Study also proved that the LBW, with premature birth (< 37 week), intrauterine growth restriction (IUGR) and other genetic factors may develop cardiovascular and renal diseases^41^. Premature birth and LBW infants are on higher risk of developing genetic congenital outcome^42^.

Children with no diarrhea are significantly less likely to be stunted and wasted both as compared to those having an episode of diarrhea and children with fever are significantly more likely to be moderately and severely wasted as compared to those having no fever. Longitudinal study has supported that the LBW newborns are more susceptible to develop fever and Diarrhoea among children under five years of age^43^. Data suggest that the infections in LBW are considered as serious condition that might increase the rate of morbidity and mortality among children^6,9,37^.

This analysis shows that female children are more affected with LBW as compared to males. Our findings were consistent with research explored that there was a higher chances of LBW among female children (OR = 1.39) as compare to male^44^. Literature also confined with our findings and proved that females are three times more likely to be stunted as compare to male child because of multiple reasons^34^. In other study LBW prevalence in female neonates were observed (8.9%) as compared to male (5.3%) and was statistically significantly (*p* = 0.0002)^45^. Similar findings were seen in a hospital based study shows a majority of female (18%) babies were born with low gestational weight as compare to male (13%), which is matching our study results^46^. Low birth females are on high risk to get diseases later in her life and proved by study that LBW is strongly associated with diabetes in women as compare to men^47^.

Strong correlation was found between LBW and malnutrition in this study among the children under five in Pakistan and moderate wasting group was found (26%) of LBW as with normal birth weights even after controlling for other factors. Hence, this shows that LBW newborns have a tendency to remain underweight in their childhood life. These findings are consistent with similar studies conducted in other parts of the world^48^. Study show that the LBW is associated with long-term health consequences has been shown with significant correlation between LBW and malnutrition and child mortality^49^.

## Limitations

The study limitations include MICS Sindh 2014 data is provincially representative data although the findings of study should be interpreted with cautions and cannot be generalizable to Pakistan as a whole. Furthermore, we did not have the data for mother height and weight hence this study remains silent on mother’s health and its role in determining the persistence of wasting and stunting in children. Of note, this study is important as recently Sindh has got a World Bank funding for Accelerated Action plan for reduction of stunting and malnutrition (AAP) and hence provide an input to this process of understanding the prevalence of stunting, wasting and their relation with low birth weight in Sindh province of Pakistan. The MICS uses mother recall regarding size of birth for determination of birth weight because many infants are not weighed at birth and those who are weighed may be a biased sample of all births, the reported birth weights usually cannot be used to estimate the prevalence of low birth weight among all children. Therefore, the percentage of births weighing below 2500 g is estimated from two items in the questionnaire: the mother’s assessment of the child’s size at birth (i.e., very small, smaller than average, average, larger than average, very large) and the mother’s recall of the child’s weight or the weight as recorded on a health card if the child was weighed at birth. Hence it may affect the correlation of LBW with other factors.

## Conclusions

In conclusion, PSM methods used for estimating treatment effects from observational data found reliable evidence. The current study investigates the impact of LBW on the child health outcomes particularly stunting and wasting using PSM method. The PSM is preferred over the conventional regression analysis because, conventional regression-based estimation, may be prone to selection bias because of the nature of non-experimental data set. Moreover, the PSM eliminates the linearity assumptions and exhibits more empirical power than conventional regression models. This study has concluded that the children with LBW are more prone to develop moderate wasting, severe wasting and both wasting and stunting among children under five years of age in Sindh Province of Pakistan. It is therefore mandatory that mother care before and during pregnancy as well as in the first 1000 days are important time for intervention to achieve the goal of undernutrition reduction in children.

## Supplementary Information

Supplementary Information.

## Data Availability

MICS data set is publically available online on the following link: http://mics.unicef.org/surveys.
