# Supplementary material for: Impact of children born with low birth weight on stunting and wasting in Sindh province of Pakistan: a propensity score matching approach
Source: Sci Rep. 2021 Oct 7;11:19932. doi: 10.1038/s41598-021-98924-7 (PMC8497567; doi:10.1038/s41598-021-98924-7)
Supplement: Supplementary file 1 — Supplementary Information. [file 41598_2021_98924_MOESM1_ESM.docx]

**Appendix-A:** Difference between the treated (LBW) and untreated groups (NBW) covariates

| Variables | Complete Sample | | | Sub Sample (Female) | | | Sub sample (Male) | | |
| --- | --- | --- | --- | --- | --- | --- | --- | --- | --- |
|  | *LBW* | *NBWl* | *Diff.* | *LBW* | *NBW* | *Diff.* | *LBW* | *NBW* | *Diff.* |
| Age | 2.66 | 2.79 | -0.088 | 2.67 | 2.78 | -0.074 | 2.65 | 2.80 | -0.103 |
| Gender | 0.49 | 0.52 | -0.057 |  |  |  |  |  |  |
| Birth order | 0.20 | 0.19 | 0.025 | 0.18 | 0.20 | -0.048 | 0.22 | 0.18 | 0.096 |
| Diarrhea | 0.37 | 0.33 | 0.086 | 0.36 | 0.33 | 0.069 | 0.38 | 0.34 | 0.101 |
| Ill with fever | 0.51 | 0.45 | 0.119 | 0.53 | 0.47 | 0.128 | 0.49 | 0.43 | 0.114 |
| Lady Health worker Visit | 0.78 | 0.77 | 0.029 | 0.78 | 0.77 | 0.023 | 0.78 | 0.77 | 0.035 |
| ANC | 0.77 | 0.77 | -0.011 | 0.78 | 0.77 | 0.023 | 0.75 | 0.77 | -0.042 |
| Hospital Delivery | 0.41 | 0.44 | -0.057 | 0.42 | 0.44 | -0.027 | 0.40 | 0.44 | -0.086 |
| Tetanus injection | 0.54 | 0.54 | 0.005 | 0.57 | 0.54 | 0.063 | 0.52 | 0.54 | -0.051 |
| Mother age | 2.77 | 2.82 | -0.080 | 2.78 | 2.83 | -0.088 | 2.77 | 2.81 | -0.071 |
| Mother education | 1.60 | 1.76 | -0.133 | 1.63 | 1.73 | -0.081 | 1.57 | 1.79 | -0.187 |
| Household size | 2.94 | 2.92 | 0.024 | 2.93 | 2.88 | 0.048 | 2.95 | 2.95 | -0.004 |
| Sanitation | 0.54 | 0.59 | -0.104 | 0.56 | 0.58 | -0.052 | 0.52 | 0.60 | -0.156 |
| Ethnicity | 2.64 | 2.64 | 0.000 | 2.60 | 2.63 | -0.030 | 2.68 | 2.65 | 0.028 |
| Wealth index | 1.46 | 1.58 | -0.154 | 1.48 | 1.56 | -0.100 | 1.44 | 1.59 | -0.208 |
| Region | 0.36 | 0.37 | -0.026 | 0.38 | 0.36 | 0.034 | 0.34 | 0.39 | -0.086 |
| Division | 2.61 | 2.75 | -0.111 | 2.63 | 2.75 | -0.091 | 2.59 | 2.76 | -0.131 |
